# Plastic Fly: What *Drosophila melanogaster* Can Tell Us about the Biological Effects and the Carcinogenic Potential of Nanopolystyrene

**DOI:** 10.3390/ijms25147965

**Published:** 2024-07-21

**Authors:** Massimo Aloisi, Daniela Grifoni, Osvaldo Zarivi, Sabrina Colafarina, Patrizia Morciano, Anna Maria Giuseppina Poma

**Affiliations:** 1 Department of Life, Health and Environmental Sciences, University of L’Aquila, 67100 L’Aquila, Italy; massimo.aloisi@studenti.unite.it (M.A.); daniela.grifoni@univaq.it (D.G.); osvaldo.zarivi@univaq.it (O.Z.); sabrina.colafarina@univaq.it (S.C.); patrizia.morciano@guest.univaq.it (P.M.); 2 INFN Laboratori Nazionali del Gran Sasso, Assergi, 67100 L’Aquila, Italy

**Keywords:** nanopolystyrene, carcinogenic nanoplastics, genotoxicity, in vivo models, *Drosophila melanogaster*

## Abstract

Today, plastic pollution is one of the biggest threats to the environment and public health. In the tissues of exposed species, micro- and nano-fragments accumulate, leading to genotoxicity, altered metabolism, and decreased lifespan. A model to investigate the genotoxic and tumor-promoting potential of nanoplastics (NPs) is *Drosophila melanogaster*. Here we tested polystyrene, which is commonly used in food packaging, is not well recycled, and makes up at least 30% of landfills. In order to investigate the biological effects and carcinogenic potential of 100 µm polystyrene nanoparticles (PSNPs), we raised Oregon [R] wild-type flies on contaminated food. After prolonged exposure, fluorescent PSNPs accumulated in the gut and fat bodies. Furthermore, PSNP-fed flies showed considerable alterations in weight, developmental time, and lifespan, as well as a compromised ability to recover from starvation. Additionally, we noticed a decrease in motor activity in DNA*lig4* mutants fed with PSNPs, which are known to be susceptible to dietary stressors. A qPCR molecular investigation of the larval intestines revealed a markedly elevated expression of the genes *drice* and *p53*, suggesting a response to cell damage. Lastly, we used *warts*-defective mutants to assess the carcinogenic potential of PSNPs and discovered that exposed flies had more aberrant masses than untreated ones. In summary, our findings support the notion that ingested nanopolystyrene triggers metabolic and genetic modifications in the exposed organisms, eventually delaying development and accelerating death and disease.

## 1. Introduction

Plastic pollution is a topical global emergency, considering its increasing concentration in the environment [1]. Quantification of plastic residues in environmental matrixes can be complex, and there is still no standard procedure for conducting these measurements [2,3,4]. A new database that summarizes the results of field sampling from scientific literature is out, and it focuses on sea and ocean analysis [5]. Over 10 pieces/m^3^ the concentration is defined as very high, from 1 to 10 pieces/m^3^ high, from 0.005 to 1 pieces/m^3^ medium, low if between 0.0005 and 0.005 pieces/m^3^ and very low if under 0.0005 pieces/m^3^. The database archive is focused on macro- and micro-plastics (MPs) that can be sampled thanks to filters and specific nets. Of note, nanoplastics (NPs) are still more challenging because of their size (<100 nm). Nowadays, only few laboratories are equipped to quantify NPs concentration, but in the majority of cases, microscopy analysis is the most commonly used, even if it is not useful for large samples [6]. Informatic models were developed to try to predict global concentrations of NPs and plastic residues in general. We know that from 1950 to 2015, we produced 350 tons of plastic every year [7]. Eriksen M. et al. [8] calculated the total plastic particles floating in the oceans, deducing an amount of 170 trillion. Implementing extraction methodologies remains a fundamental aim to really understand the gravity of plastic pollution and to better design research studies. Sources of environmental pollution range from industrial production to vehicle emissions and domestic waste [9,10]. In the last category, Gui J. et al. [11] found around 2400 items/Kg of plastic in Chinese rural city waste. Depending on clothing materials, plastic residues are also released into the environment from washing machines and household appliances [12]. A sustainable textile industry is a valid choice to decrease plastic pollution. The friction of car wheels on asphalt is another source of plastic particles. Polymers from car wheels are today considered a class of plastic material based on their chemical properties [13]. Globally, a release of 0.81 kg/year of micro-gums is estimated to become an important fraction of air particulate matter [13,14,15,16]. The impact of MPs and NPs on human health is still controversial. It was seen that NPs accumulate into the cytoplasm of mammalian cells [17]. External membrane charge is crucial to the absorption process; positively charged- and uncharged particles are more easily internalized than negatively charged ones, probably because of the electric properties of cell membranes [18,19]. Particle shape is also important; indeed, MPs and NPs used in scientific works are generally spherical, but a wide spectrum of shapes can be found in the wild with different physical and aggregation properties [14,20]. The main degradation pathways from macro- to micro- and nano-plastics are induced by chemical and physical sources such as UV rays, heat, pH, and salinity through a radical-dependent process [21,22]. Biological pathways of degradation contribute either way. Increasingly, bacterial species are found living on plastic fragments, favoring their degradation [23,24]. Zooplankton can ingest them and release smaller digested plastic residues into aquatic environments [25]. Regarding in vitro studies, numerous papers are today available. Using Caco-2 cells as an intestinal model, cytotoxicity or permeability defects were not found, but only low oxidative stress [26,27,28,29]. Roursgaard et al. [30] using particles obtained from the artificial degradation of plastic bags to mimic environmental plastic mixtures, did not observe cytotoxicity either, but a reduction in lipid metabolism. Despite the fact that there was not oxidative stress, the comet assay showed an increase in double-strand breaks. In the HCT116 cell line, we observed a reduction in cell viability and micronuclei formation due to oxidative stress [31]. Caco-2 and HCT116 are the most commonly used cell lines for intestinal genotoxicity studies [32]. In addition, single-cell cultures are more affected than co-cultures or organoid cultures, showing the necessity of more complex models to really mimic human complexity [32]. Plastic residues can be found in the air too; therefore, using pulmonary cells is important to study the impact of MPs on the respiratory exposure route. Yang et al. [33] saw human pulmonary alveolar epithelial cells (HPAEpiCs) inflammation as a source of oxidative stress, alteration of the expression of genes involved in detoxification and apoptosis. Moreover, they observed a reduction in transepithelial resistance, leading to less functionality. The most common mechanisms are related to inflammation and apoptosis, especially with smaller NPs [34,35,36]. Given the relevance of MPs in cosmetic products, we also previously tested the HS27 fibroblast cell line, detecting genotoxicity and a dose-dependent reduction of cell viability [37]. A selection of in vivo studies on the main model organisms is summarized in Table 1. They allow us to study factors such as kinetics and systemic/multiorgan exposure and permit better translational results [38].

In addition, epigenetic alterations have been observed and recently reviewed in [52]. It is still a growing field of research; therefore, much more must be done, but it is relevant to understand if multi-generational and trans-generational effects are present. Scientific literature papers reported alterations in epigenes such as methyltransferases and modulation of genes involved in apoptosis, immune response, and lipid metabolism [52,53,54]. *Drosophila melanogaster* is a well-established model to study the effect of compounds, drugs, and nanomaterials on development and their genotoxic properties [55,56,57]. The fruit fly has a life cycle of around 10 days at 25 °C (from eggs to adults), and a single female lays hundreds of eggs, making it possible to follow a high number of generations and individuals. Moreover, a large number of behavioral and genetic assays are available to assess general biological effects and genotoxicity. The aim of this study is to exploit the genetic potential of *Drosophila melanogaster* to study the impact of polystyrene nanoparticles (PSNPs) in wild-type and mutant flies, detecting their biological effects, genotoxicity, and carcinogenic potential.

## 2. Results

### 2.1. Analysis of Nanopolystyrene with Scanning Electron Microscopy (SEM) 

**Figure 1:**
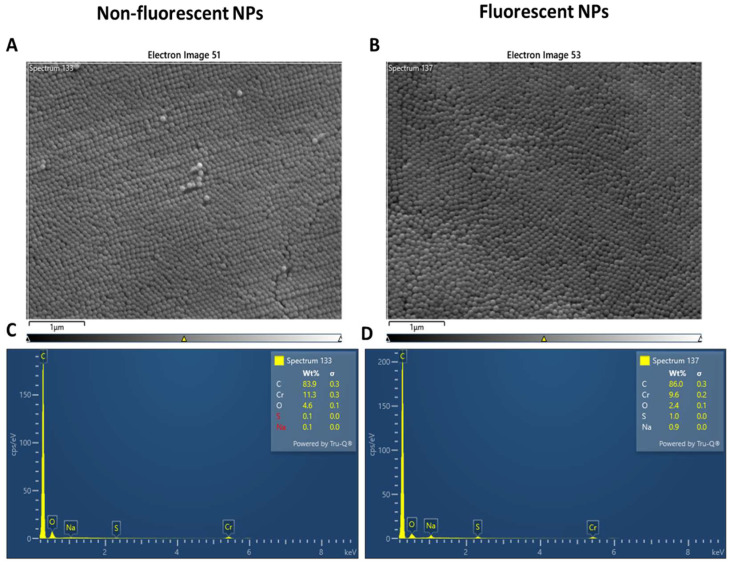
Morphological analysis (**A**,**B**) and energy dispersive X-ray analysis (**C**,**D**) of polystyrene nanoparticles, non-fluorescent (**A**,**C**) and fluorescent (**C**,**D**).

Electron microscopy was used to characterize PSNPs. PSNPs had an average size of 100 nm, according to the manufacturer’s specifications (Figure 1A,B). Energy dispersive X-ray analysis (EDX) was used to confirm the absence of metals or other contaminants. The spectrum reveals only the presence of carbon, confirming the purity of the solution and the presence of oxygen due to the presence of water residue. Chromium is added to perform SEM analysis, and Na and S are needed as preservatives (Figure 1C,D).

**Figure 2:**
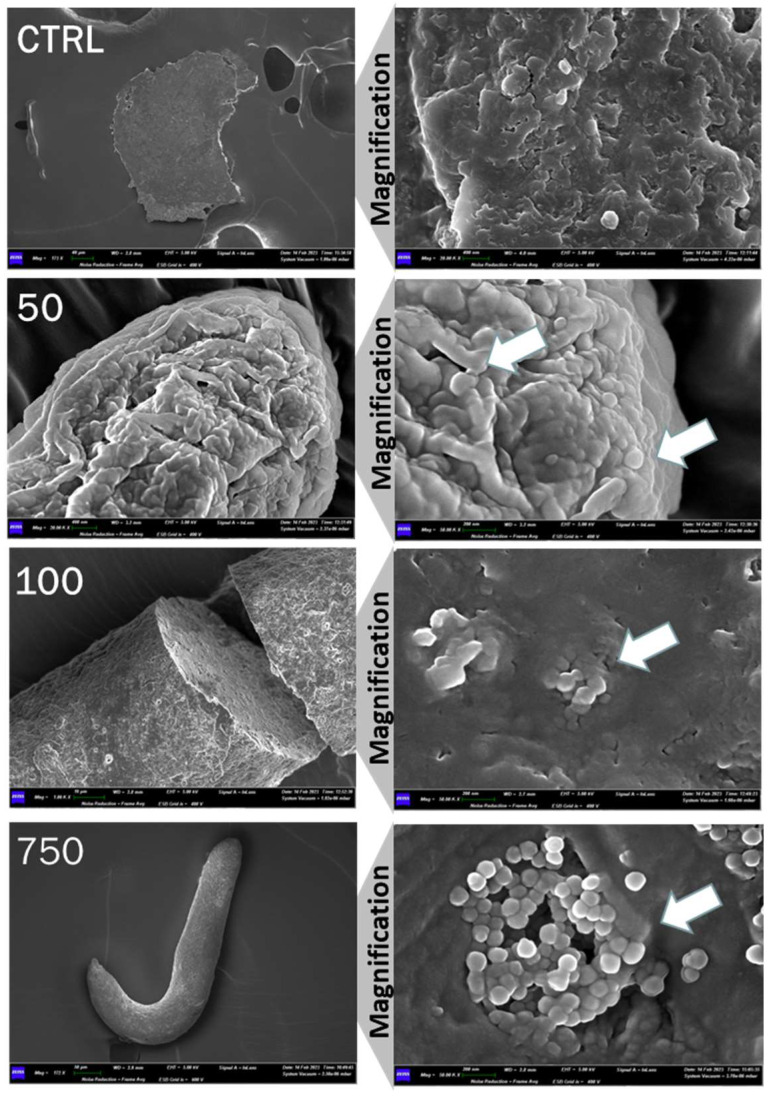
SEM analysis of PSNPs in feces from untreated flies (CTRL) and three different concentrations (50 μg/mL, 100 μg/mL, and 750 μg/mL). Left column has 172 X magnification for CTRL and 750 μg/mL, 20.00 K X for 50 μg/mL, and 1.00 K X for 100 μg/mL; the right column magnification is 50.00 K X for every condition. White arrows point to NPs.

Excrements were collected from the vial’s walls of treated and untreated wild-type flies and analyzed with SEM (Figure 2). The feces of the negative control were devoid of PSNPs, while those of the treated flies showed the presence of PSNPs averaging 100 nm, suggesting that the digestion process did not lead to an alteration of PSNPs size. At the highest concentration of PSNPs, the particles form aggregates in feces.

### 2.2. Biodistribution and Absorption

**Figure 3:**
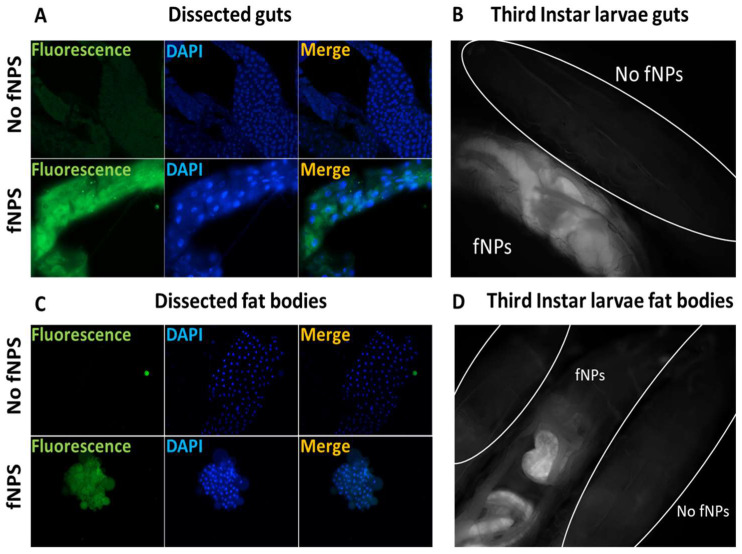
Fluorescence microscopy of fPSNPs in OR-R third instar larvae guts and fat bodies in two conditions: negative control (no fPSNPs, 0 μg/mL) and 750 μg/mL (fPSNPs). (**A**,**C**) dissected guts and fat bodies, respectively (400X); (**B**,**D**) whole living larvae (100X).

The ingestion of fluorescent PSNPs (fPSNPs) allowed the study of the biodistribution of plastics in larvae. Live and dissected *OR-R* larvae were observed under a fluorescence microscope (Figure 3). In living larvae, ingested fPSNPs are found in the gut (Figure 3B) and in body structures that are likely to be fat bodies. The analysis of dissected larvae confirms the presence of fNPs in guts and fat bodies (Figure 3A,C). The noticeable presence of PSNPs in fat bodies is probably due to the lipophilic nature of PSNPs (Figure 3D) and is indicative of the passage of PSNPs across the intestinal barrier and their release in the haemolymph (the fly blood).

### 2.3. Development Traits (Life Cycle and Eclosion)

**Figure 4:**
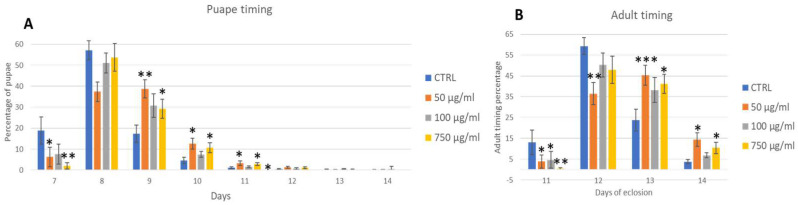
Analysis of development traits of OR-R flies: pupae formation (**A**) and adult timing (**B**). Treatments: Control (0 µg/mL); 50 µg/mL, 100 µg/mL, 750 µg/mL. * *p* < 0.05; ** *p* < 0.005; *** *p* < 0.001.

Wild-type parental flies were fed with PSNPs added to the instant food at various concentrations and left to lay eggs overnight on the PSNPs food. A significant reduction in the developmental traits of the offspring was observed. First, we assessed developmental parameters, such as the timing of pupae and adult emergence. Figure 4A,B shows the results. In untreated control, pupae begin to appear on day 7 after egg laying (AEL), and a significant reduction in pupae’s number in 50 µg/mL and 750 µg/mL conditions was found. On the 8th day, the difference disappears, even if the tendency remains, and from the 9th day on, the number of pupae increased in treatments, balancing the first-day difference. The result of this analysis thus shows a delay of 24 h in the larvae–pupae development. Adults’ emergence follows the same pattern except for 50 µg/mL, which remains significantly reduced on the second day. Interestingly, the concentration of 100 µg/mL does not affected this parameter. Eclosed adults show no differences in number (about 93% of pupae develop into adults in all conditions) or sex ratio. (Figure S1A,B in Supplementary Materials; Development traits of exposed adults). No obvious morphological defects were observed in adults.

### 2.4. Weight

**Figure 5:**
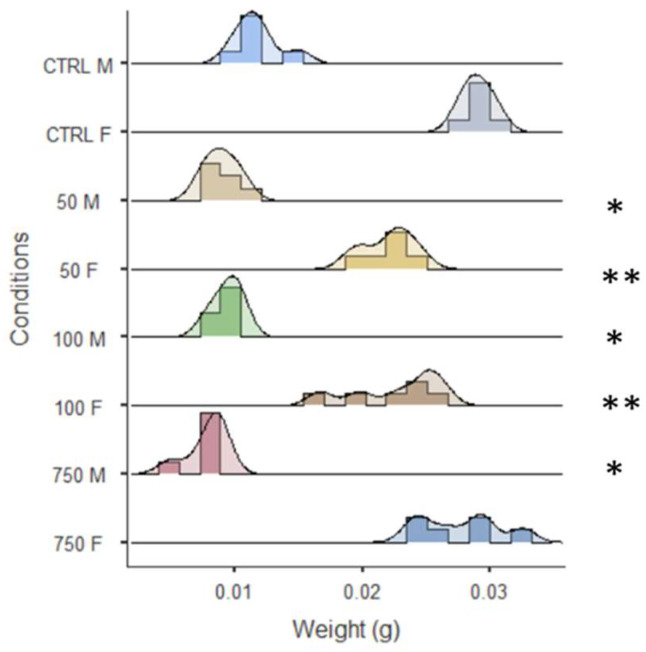
Weight distribution of 3 days old OR-R flies. * *p* < 0.05; ** *p* < 0.005.

Considering the delay in larvae–pupae development, the weight of adults was investigated. Figure 5 shows that the weight of treated male and female adults was significantly reduced with respect to the untreated flies.

### 2.5. Stress Response 

**Figure 6:**
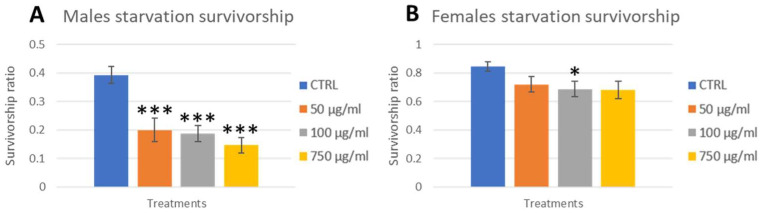
Starvation assay with OR-R flies. Three days-old adults chronically exposed were starved in empty vials and checked hourly for the first 12 h (Figure S2A,B Supplementary Materials) and after 24 h (**A**,**B**). Treatments: CTRL (0 µg/mL); 50 µg/mL; 100 µg/mL; 750 µg/mL. * *p* < 0.05; *** *p* < 0.001.

To assess if the developmental delay and reduced weight had any effects on adult fitness, we exposed flies to different secondary stresses (after PSNPs ingestion): starvation and exposure to high temperatures (seizure test). For the starvation assay, previously treated flies were left without food in empty vials for 24 h and scored for mortality every hour for the first 12 h (Figure S2A,B in Supplementary Materials; Starvation in exposed adults) and finally after 24 h (Figure 6A,B). No significant differences were observed during the first 12 h. After 24 h, males exposed showed significantly higher mortality at all concentrations. Females also showed a clear, although not significant, tendency to be less resistant to starvation at all concentrations.

For the seizure test, we exposed flies to 42 °C for 2 min and evaluated the number of fainting flies and recovery time. No significant differences were observed, suggesting the nervous system be not affected by PSNPs under our conditions. 

### 2.6. Larval Crawling and Wall Body Contraction

A larval crawling assay was performed on third instar OR-R larvae. No significant differences were found in terms of length of the crawls performed within a given time and body contractions per minute (Figure S3 in Supplementary Materials; crawling assay and wall body contraction screening in wild-type third instar larvae fed with PSNPs). These data showed that PSNPs do not affect movement ability or larval muscle functionality.

### 2.7. Adults Climbing

**Figure 7:**
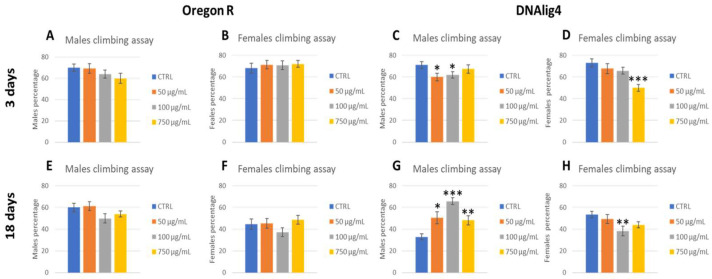
Climbing assay of OR-R (**A**,**B**,**E**,**F**) and DNA*lig4* (**C**,**D**,**G**,**H**) flies after 3 (**A**–**D**) and 18 (**E**–**H**) days from eclosion, divided into males (**A**,**C**,**E**,**G**) and females (**B**,**D**,**F**,**H**). * *p* < 0.05; ** *p* < 0.005; *** *p* < 0.001.

A climbing assay on wild-type and *lig4* mutant adults was performed. DNA ligase 4 is a factor involved in DNA damage response and specifically catalyzes the final step of DNA end ligation in the NHEJ pathway [58]. In *Drosophila, lig4* mutants are viable and sensitive to ionizing radiation during embryonic development. Interestingly, recently, *lig4* mutant adults were found to be sensitive to nutrient stress, suggesting that *lig4* is required for maintaining health and longevity in *D. melanogaster* [59]. The *lig4* mutant was used to evaluate the effects of PSNPs in an already known stress-sensitive background. The assay was carried out on 3 days- (Figure 7, upper panel) and 18 days-old (Figure 7, lower panel) flies to evaluate the contribution of PSNPs toxicity to aging. While in OR-R no difference was found in the flies’ climbing ability, in *lig4* mutants, after an initial decrease in climbing performance in young adults, the 18 days old male treated flies maintained higher locomotor activity than untreated flies. Note that the two age groups consisted of the same flies that were constantly maintained on food mixed with PSNP. Dead flies were recorded. In *lig4* mutants, 3% control males, 13% 50 µg/mL, 4% 100 µg/mL, 1% 750 µg/mL, and 5% control females, 1% 50 µg/mL, 5% 100 µg/mL, and 10% 750 µg/mL, were found dead after 18 days. No OR-R flies died.

### 2.8. Trypan Blue and Smurf Assay

**Figure 8:**
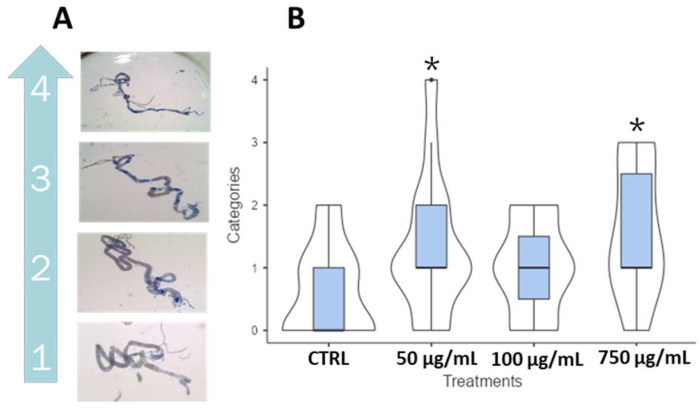
Trypan blue assay in wild-type third instar larvae gut fed with PSNPs. (**A**) dissected guts were incubated with Trypan blue and then scored under a stereoscope based on blue intensity and spreading; (**B**) violin plot and box plot showing the final scoring of the four conditions used (0, 50, 100, and 750 μg/mL). * *p* < 0.05.

The Trypan Blue test was performed on third instar wild-type larvae to assess potential intestinal damage following PSNPs ingestion. Based on the extent of blue staining, the dissected guts were classified into five classes, from 0 to 4 (Figure 8A). A significant, though not drastic, increase in cell death was found at concentrations of 50 μg/mL and 750 μg/mL. To evaluate gut damage in adults, the Smurf assay was performed on *w*^1118^ flies fed with PSNPs. No significant results were recorded, but an interesting trend of *smurf* flies was found in both males and females, increasing with concentrations (Figure 8B). Considering the results, we continued to investigate the impact of PSNPs specifically on larval guts with the comet assay and qPCR on apoptotic gene expression.

### 2.9. Pro-Apoptotic Gene Expression

**Figure 9:**
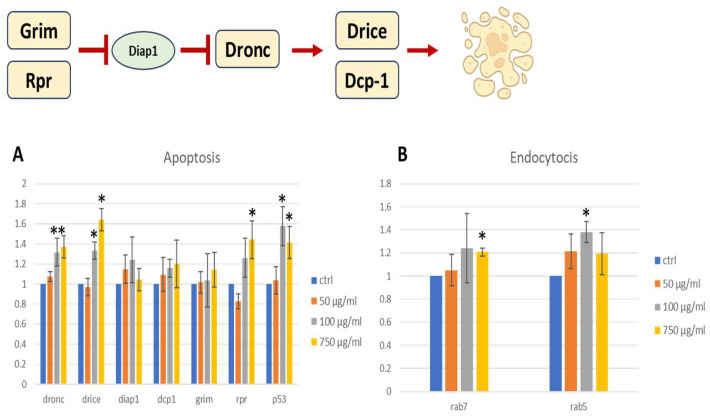
A schematic drawing of the apoptotic process. Image realized with Biorender.com. Gene expression of apoptosis (**A**) and endocytosis (**B**) biomarkers in third instar larvae guts. Student’s *t* test was employed to compare the values of untreated (ctrl) and treated. * *p* < 0.05; ** *p* < 0.005.

Given the presence of larval intestinal damage detected by the Trypan blue assay, a qPCR was carried out to evaluate the expression of apoptotic genes in larval guts. Caspases are the main executors of the apoptosis process. Important factors for the apoptotic response are Dronc (a caspase-9-like initiator caspase), Dcp-1, and Drice, which are caspase-3-like effector caspases. In the absence of apoptotic signals, inhibitors of apoptosis proteins (IAPs), such as Diap1, bind to caspases and inhibit their activity. In the presence of apoptotic stimuli, the pro-apoptotic proteins Reaper (Rpr), Hid, and Grim bind to Diap1 and release the caspases [60]. A schematic and simplified drawing of the described apoptotic process is shown in Figure 9. Another important player in the apoptotic process is the nuclear transcription factor p53, which regulates the expression of genes involved in apoptosis as well as growth arrest and senescence in response to genotoxic or cellular stress [61]. In our qPCR analysis, the expression of *dronc*, *drice*, *diap1*, *dcp1*, *grim*, *rpr*, and *p53* was evaluated in larval guts.

Expression of the pro-apoptotic genes *rpr*, *drice*, and *dronc* was significantly increased in two concentrations (100 and 750 μg/mL) compared to the negative control, indicative of the presence of an apoptotic stimulus, as confirmed also by the higher expression of *p53*, a cellular stress response gene. The apoptotic stimulus detected is probably consequent to gut damage shown in Figure 8. The PSNPs also seem to stimulate endocytosis, as shown by the upregulation of the two markers of the endolysosomal pathway, *rab5* and *rab7*, in treated flies. Rab5 is a marker for early endosomes, replaced by Rab7 during maturation [62]. Although only the highest concentrations of PSNP result in significant differences, there is a general trend toward an increased expression of endocytic markers in all the treated samples, indicating that transmembrane transport may participate in PSNP uptake.

### 2.10. Comet Assay

**Figure 10:**
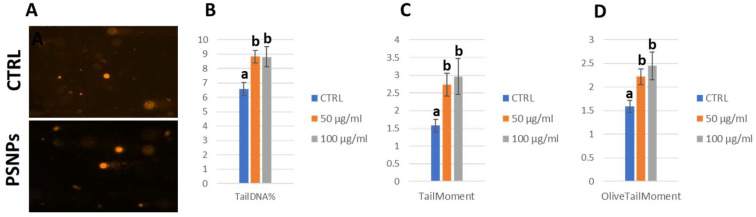
Dissected *OR-R* third instar larvae guts were homogenized to obtain a cell suspension that was subjected to an electrophoretic run and subsequent nuclear stain with ethidium bromide to observe DNA breaks under a fluorescence microscope (**A**). The percentage of DNA detected in comet tails, the tail moment and the olive tail moment are reported (**B**–**D**). Non-parametric one-way ANOVA was used for significance analysis. Non-significant conditions were grouped with the same letter; different letters represent significance between conditions. Treatment: Control (0 µg/mL); 50 µg/mL; 100 µg/mL.

To further confirm that pro-apoptotic stimulation and overexpression of p53 in larval guts were associated with DNA damage, we carried out a comet assay in these tissues (Figure 10A). Over the past few years, comet assays have been successfully used in *Drosophila* to study genotoxicity as well as DNA repair [63]. For this analysis, we focused on the lower concentrations, 50 μg/mL and 100 μg/mL.

We scored specifically three indexes: tail DNA%, tail moment, and olive tail moment. Both concentrations were significant compared with the negative control, confirming the presence of DNA damage in gut cells. All indexes were significantly higher in treated flies with respect to the control (Figure 10B–D).

### 2.11. Carcinogenic Potential

**Figure 11:**
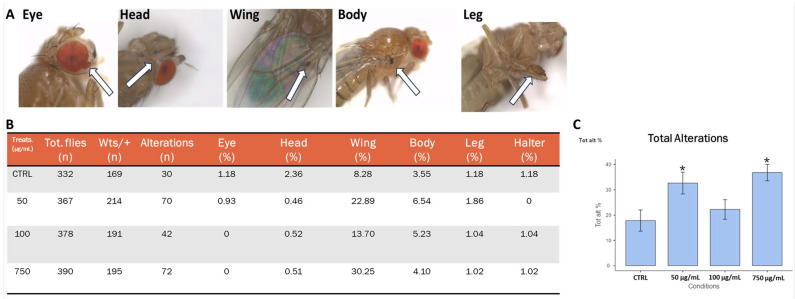
*warts* assay showing carcinogenic potential of PSNPS. (**A**) representative images of tumors in 3 days old *wts* defective flies (white arrows); (**B**) table resuming the percentage of masses in the *wts* fly population; (**C**), bar plot showing the percentage of total alterations. Treatment: CTRL (0 µg/mL); 50 µg/mL, 100 µg/mL, 750 µg/mL. * *p* < 0.05.

To assess if PSNPs can favor carcinogenesis, we used genetically predisposed *warts* mutant flies and exposed them to 0 µg/mL, 50 µg/mL, 100 µg/mL, and 750 µg/mL of PSNP. *warts* encodes for a tumor suppressor factor of the Hippo pathway involved in tissue growth control. In *Drosophila*, mutations in the *wts* gene are recessive lethal, while viable heterozygous adults occasionally show large tumorous outgrowths in their bodies. This genetic background has been used to evaluate the carcinogenic potential of several compounds and can be used in toxicological studies. Heterozygous *wts*/+ flies were selected and scored for tumoral masses after PSNPs exposure (Figure 11A,B). As shown in Figure 11C, in two concentrations, we observed an increased number of abnormal masses, except for the concentration of 100 µg/mL.

## 3. Discussion

Plastic pollution is increasing every year and is considerable both in the oceans, seas, freshwater lands, and the atmosphere [64,65]. Chemical and physical factors can induce its degradation, leading to the formation of micro- and nano-fragments that multiply its toxicity on different scales that need to be deepened [66]. Their presence affects organisms, inducing oxidative stress, inflammation, fibrosis, and altered metabolism [67,68,69,70]. In this study, we decided to focus on polystyrene nanoparticles (PSNPs) for several reasons. PSNPs are one of the most prevalent NPs in the environment and are widely used in food packaging, posing a hazard to humans [71].

In our study, we used *Drosophila melanogaster* to study the impact of PSNPs exploiting their genetic similarity with humans and their well-recognized usage for biomedical research [54,55,56,57]. Initially, an overall characterization of PSNPs confirmed their dimensions and chemical composition. The PSNPs were administered to flies by ingestion mixed with standard fly food. Three different concentrations were used in the study based on previous papers found in the literature and our published data [31,37]. Parental individuals were crossed in the presence of PSNPs, and the laid embryos developed into adults on contaminated food to determine chronic exposure throughout development. In larvae, the distribution of fluorescent NPs indicates that PSNPs cross the intestinal barrier as they were found abundantly in fat bodies, perhaps as a consequence of their lipophilic nature. Such crossing is at least partly attributable to an endocytosis process, as suggested by the expression of endocytosis-related genes in this tissue. No fluorescence was evident in other organs.

Chronically exposed individuals showed increased pupal development time and adult emergence of about 24 h compared to control. Tu et al. [72] found a similar developmental retardation, but in later generations. 

We can assume that PSNPs physically reduce the amount of actual ingested food, limiting storage. This can be confirmed by the fact that treated flies have a significant reduction in weight. Also, the trapped PSNPs in the fat bodies could provide a “second hit” when flies try to use their lipid reservoir. In fact, treated flies deprived of food showed less resistance to starvation, probably for the previous reasons. It can be concluded that the ingestion of NPs results in a general alteration of metabolism that leads to a fitness reduction in *Drosophila*.

Exposure to PSNPs in both wild-type larvae and adults showed no adverse effect on their movement ability. In contrast, Kauts et al. (2023) [73] found a reduction in larval crawling and adult climbing ability in the wild type, but using a different type of NPs, PET. Concerning the *DNAlig4* mutants, we observed that aged flies exposed to PSNPs, after an initial decrease in their climbing performance, seemed to rescue their ability, surpassing untreated flies. Similar favorable results were obtained by Liang and colleagues [74], who studied the impact of PET microplastics of 2 µm on the lifespan of wild-type adult males. They observed that exposure to PET at a concentration of 1 g/L induced a slight increase in lifespan. This result is explained by the authors with the hormetic phenomenon that consists in the development of a positive response in individuals exposed to mild stress. It was already demonstrated with low dose radiation, heat shock, and hypergravity [75,76,77] and depends in part on DNA repair factors like ATR, ATM, and stress signal transducers (Sir2/SIRT1, JNK, p53, and dFOXO) in flies [78]. Moreover, in flies, the radioadaptive response, which is closely related to hormesis, relies on LIG4 [79]. For these reasons, in our experiment, it is difficult to assume that the beneficial effect observed in *lig4* mutants can be attributed to hormesis. We can speculate that exposure to NPs increased the susceptibility of the *lig4* mutants to mortality and resulted in the selection of the strongest individuals. The climbing test at 18 days was carried out on the *lig4* flies that remained alive and resulted in better motor activity than the untreated control that did not undergo such selection. More studies are needed to understand this interesting phenomenon. 

While adult guts showed only a light tendency to be affected by PSNPs, larvae showed a significant presence of cell death in the intestine. That prompted us to verify how PSNPs administration was translated into an apoptotic stimulus by evaluating the modulation of some genes associated with cellular death. We observed a significant increase in *rpr* expression, which is an initiator of apoptosis, and in *dronc* and *drice*, which actuate apoptosis [80,81]. Moreover, there was a significant increase in the *p53* transcript, a cellular stress response protein, confirming the presence of some kind of perturbation in the gut and suggesting an apoptotic response. Future biochemical analysis is needed to verify whether modulation of gene expression results in modulation and/or activation at the protein level. A comet assay on larval guts showed a significant amount of DNA damage, possibly related to the cell death detected. Alaraby et al. [42] also observed increased DNA damage with the comet assay linked to oxidative stress in larvae using a true-to-life solution of PET nanoplastics. They used hemocytes, assessing that once absorbed, nanoplastics can induce DNA damage in other cellular types as well.

To assess the carcinogenic potential of PSNPs, we used mutants defective in *warts*. Warts is a serine/threonine kinase that phosphorylates Yorkie, thus controlling organ growth and cellular proliferation downstream of the Hippo pathway [82]. *wts* heterozygous flies develop melanotic masses spreading throughout their bodies, making them a suitable model to study the impact of the environment on cancer-predisposed individuals [83]. We found an increased number of tumoral masses, showing that, although wild types did not show any phenotypic anomalies, a predisposed organism can be more susceptible, as in the case of cancer. A possible explanation for the increased presence of tumors in adult *warts* heterozygote mutants could be LOH as a consequence of the DNA damage detected by the comet assay in larval gut tissue that contributes to genome instability [84].

In general, we found that male flies were more susceptible than females to NPs exposure. Sex-related differences were also found by Urbisz and colleagues [85] who observed histological alterations in the midgut that were more prominent in males after exposure to micro- and nano-polystyrene particles. This sex-dependent dimorphic response could be a consequence of the fact that some genes related to stress response evade the dosage compensation effect, resulting in more expression in females [86]. More generally speaking, several significant differences have been observed between *Drosophila* males and females in the regulation of metabolism, in response to nutritional challenges, and in immune response. This sex diversity is expected to result from differences in gene expression. However, the sex-specific differences are still underestimated and therefore poorly understood [87].

Differences in biological responses between sexes were also observed in other organisms. For instance, in the crustacean *Macrobrachium nipponense*, PSNPs of 75 nm can induce an initial increase followed by a decrease in sex hormones (estradiol, progesterone, and testosterone), causing alterations in the expression of sex-related genes in the gonads [88]. Moreover, in zebrafish, the co-exposure of 50 nm PS and triclosan, which is an antimicrobial agent found in personal care products, induced opposite effects in the two sexes. In males, the authors observed an increased presence of triclosan in the testes, while in females, the amount in the ovaries decreased. Following an opposite trend, male’s genes involved in sex-hormones decreased while females increased [89].

In conclusion, our findings showed that ingested PSNPs trigger metabolic and genetic modifications in the exposed organisms, eventually delaying development and accelerating death and disease. Moreover, we observed that the presence of mutations can induce different results, suggesting the genetic background may be relevant in determining how an organism responds to PSNPs exposure.

## 4. Materials and Methods

### 4.1. Flies Husbandry and Nanopolystyrene Exposure

All of the strains were obtained from *Bloomington Stock Center* and maintained at a constant temperature with a relative humidity of approximately 60%, a temperature of 25 °C, and fed with standard food (Nutri-Fly^®^ Bloomington Formulation). In particular, the following strains were used: *Oregon R (OR-R)*, *w*^1118^, *Lig4*^5^, and *st,in,kni,p,wts*^3–17^/*TM3, Sb* mutants. The fruit flies were cultured in the dark. *warts* mutant was kindly sent by Dr. Fiammetta Vernì from Sapienza University (Rome, Italy).

For all the treatments, flies were kept at 25 °C and fed with instant food (Flystuff Nutri-Fly Food, Instant Formulation. This formulation requires no heating for preparation) with or without polystyrene nanoparticles (PSNPs) (oral exposure) and is allowed to lay eggs overnight in experimental vials. The resulting F1 generation was used as third-instar larvae or 3- and 18-days old adults. Concentrations: negative control (0 µg/mL); treatments: 50 µg/mL; 100 µg/mL; 750 µg/mL of PSNPs.

### 4.2. Poyistyrene Nanoparticles

Fluorescent (f) and non-fluorescent PSNPs of 100 nm of diameter were purchased from Polysciences Europe GmbH Badener Str. 13 69493 Hirschberg an der Bergstrasse Germany (fPSNPs; Fluoresbrite^®^ Yellow Green Microspheres; cod. 17150-10; 0.10 µm 2.5% particles in water solution; 4.55 × 1013 particles/mL; Excitation max = 441 nm; Emission max = 486 nm; PSNPs; cod. 00876-15; 0.10 µm 2.5% particles in water solution; 4.55 × 1013 particles/mL).

### 4.3. Scannng Electron Microscopy and EDX Analysis

To characterize PSNPs and wild-type excrements, we used scanning electron microscopy (SEM) (Gemini Field Emission SEM 500, ZEISS, Milan, Italy) equipped with an X-ray microanalysis system (EDS Oxford Inca 250 x-act). The first one was used to assess PSNPs morphology from the stock solution to flies’ feces; the second one was used to conduct an elemental analysis of the particles. For SEM analysis, 1 µL of particle sample was deposited on a stub and left dehydrated in air; then, a 5 nm film of chromium was deposited on it with Sputter Quorum 150T ES to make it conductive. Flies’ excrements were collected from vial walls, left to dry in the air, and then the same protocol used for particles was followed. We used SEM analysis to obtain the morphological and chemical details of our samples.

### 4.4. Fluorescence Microscopy

To deepen PSNPs distribution in third instar larvae, we used fluorescence microscopy (ZeissAxio Imager M2) on living and dissected larvae. Third instar larvae were washed three times in PBS to remove external residues of food and then observed under the microscope. To assess if fPSNPs were absorbed by intestinal cells and bioaccumulated into other organs, we washed the larvae three times, dissected guts and fat bodies in a 0.7% physiological solution, fixed them in 4% formaldehyde, counterstained them with DAPI, and placed them on microscope slides for the observations.

### 4.5. Development Traits and Weight

To assess if PSNPs have an influence on the development of OR-R flies, we scored the timing of pupae formation and adult eclosion, viability, and sex differences. To do so, we counted every day at the same hour the number of pupae and adults, dividing them into males and females. The experiment was repeated 12 times (around 1200 pupae and adults scored). Adults were also observed under a stereomicroscope to detect eventual abnormalities in the eye, wings, and whole body. For weight, 100 3 days old OR-R adults were collected and divided into two groups of 50 flies, males and females, for three experiments (300 males and 300 females for each concentration).

### 4.6. Stress Tolerance

To evaluate if PSNPs exposure can influence flies’ survival mechanisms, we exposed non-treated and treated (0, 50, 100, and 750 μg/mL) OR-R flies to starvation and heat. For the starvation, we followed the Shaposhnikov protocol with some modifications [90]. Briefly, 3 days old flies were collected, split into males and females, and grouped ten by ten in empty vials. The assay was performed three times with 50 males and 50 females each (300 flies in each concentration). We scored the number of dead flies every hour for the first 12 h and then after 24 h. For the heat-shock assay (seizure assay), we followed the Mituzaite protocol with some modifications [91]. Briefly, 3 days old flies were collected and grouped for the starvation assay. The experiment was performed three times with 30 males and 30 females each (180 flies for every concentration). Every vial was immersed in water at 42.5 °C for 2 min, scoring how many flies faint every 20 s. Then, we scored the timing of recovery and how many flies needed more than 4 min to do so.

### 4.7. Climbing Assay 

We performed a standard climbing assay on OR-R and *DNAlig4* defective mutants following the Iuso et al. protocol with some modifications [92]. We collected 3- and 18-days old individuals and divided them into males and females. The experiment with the OR-R was repeated three times, scoring 50 males and 50 females each (300 flies for every concentration) grouped ten by ten in empty vials. The *DNAlig4* experiment was repeated six times, scoring 50 males and 50 females each (600 flies in total). The assay consisted in measuring how many flies could reach a height of 7 cm in 20 s after being tapped down. The 3 days old and the 18 days old flies were the same; eventually, dead individuals were scored too.

### 4.8. Larval Crawling Assay and Wall Body Contraction 

Wild-type larvae mobility was assessed by comparing treated and non-treated individuals. We used four conditions: 0, 50, 100, and 750 μg/mL. For the larval crawling assay, we followed Nichols and colleagues [93] protocol with some variations. A total of 36 OR-R larvae for each PSNPs concentration (three experiments, 12 each) were placed on a Petri dish with 1.5% agarose gel. We tracked the distance crossed per minute with graph paper. For the wall body contraction assay, the same number of larvae were placed singularly in physiological solution drops with a little bit of fresh waste. Then, we scored the number of contractions per minute under a stereomicroscope. 

### 4.9. Smurf Assay (Intestinal Integrity Test)

To gain insight into gut damage and barrier dysfunction in adults, we performed a Smurf assay [94]. The experiment was repeated three times, dividing males and females with around 107 flies per concentration. Three days old *w*^1118^ flies grown in four different concentrations (0, 50, 100, and 750 μg/mL) were left on food added with FCF Brilliant blue for 24 h. Then, under a stereomicroscope, they were scored in no smurf and smurf based on the absorption of the dye. The *w*^1118^ flies were used because they are characterized by a lighter color of the abdomen, which allows a better view of the intestine [95].

### 4.10. Trypan Blue Assay

We performed the Trypan blue assay as a first sight into gut damage in larvae. We followed the Carmona et al. (2015) protocol with some light modifications [96]. Wild-type third instar larvae grown in four concentrations (0, 50, 100, and 750 μg/mL) were collected and washed three times in PBS. Then, guts were dissected, fixed in 4% formaldehyde, and stained in a 0.02% Trypan blue solution (diluted in PBS) for 30 min at 25 °C. A total of 15 guts per concentration were scored (5 per replicate). Finally, guts were placed on microscope slides and scored into five categories [96]: “no color = 0; light blue = 1; darkly stained nuclei = 2; large patches of darkly stained cells = 3; or complete staining of most cells in the tissue = 4”.

### 4.11. Total RNA Extraction and RT-qPCR

Variations in gene expression in treated and non-treated OR-R third instar larvae guts were evaluated by qPCR. We analyzed apoptosis-related genes (*hid*, *rpr*, *dronc*, *drice*, *p53*, *diap1*, and *dcp1*) and endocytosis-related genes (*rab5* and *rab7*). Larvae were collected and washed three times in PBS; then, we dissected 40 guts per concentration. Total RNA was isolated from samples using TRIzol (Thermo Fisher Scientific, Waltham, MA, USA, Cat. 15596026) according to the manufacturer’s instructions.

The integrity of the RNA was immediately checked using 1.2% agarose gel electrophoresis (1 µg samples). The RNA concentration was assessed by the Qubit dsDNA BR Assay Kit (Invitrogen, Life Technologies, Carlsbad, CA, USA, cat. Q32850) used for the quantification of cfDNA on the Qubit 3.0 Fluorometer (Invitrogen, Life Technologies, Carlsbad, CA, USA), and RNA samples were stored at −80 °C until use.

First-strand cDNA synthesis was performed from 1 µg of total RNA using the Maxima H Minus First Strand cDNA Synthesis Kit with dsDNase (Thermo Fisher Scientific, Waltham, MA, USA, cat. K1681) according to the manufacturer’s instructions. The dsDNase enzyme was used in the first step to remove any contamination of the genomic DNA; at the same time, a no-RT control reaction was carried out.

RT-qPCR was performed in a final volume of 20 µL, including 15 ng of the cDNA product, specific forward and reverse primers (500 nM), and PowerUp SYBR Green Master Mix (Applied Biosystems cat. A25741, Waltham, MA, USA), which is a ready-to-use cocktail containing all components (including ROX Reference Dye at a final concentration of 500 nM).

The amplification reaction was performed in an Applied Biosystems 7300 system (ThermoFisher Scientific, Rockford, IL, USA), and all the PCRs were performed under the following conditions: 2 min at 50 °C, 2 min at 95 °C, and 40 cycles of 15 s at 95 °C and 1 min at 60 °C in 96-well optical reaction plates (Applied Biosystems, Waltham, MA, USA). The specificity of the qRT-PCR reactions was monitored through melting curve analysis (60–95 °C), after 40 cycles.

The relative expression ratio was analyzed using a delta–delta Ct (2^−ΔΔCt^) method [97]. *actin* and *α tubulin* were chosen as the reference genes, and the control was used as the calibrator. Three biological replicates were performed, and all samples were carried out in duplicate.

The genes analyzed and NCBI Reference Sequence were hid (NT_037436.4), *rpr* (NT_037436), *dronc* (NT_037436), *drice* (NT_037436), *p53* (NT_033777.3), *diap1* (NT_037436), *dcp1* (NT_033778), *rab5* (NT_033779), and *rab7* (NT_033779). The primer sequences used in this study are listed in Table S1 in the Supplementary Materials (Sequence of the primers used for the qPCR analyses). Some primers were found in the literature, others were constructed on gene sequences with Primer Express 3.0 software (Applied Biosystems, USA); primers were synthesized and bought from Eurofins Genomics (Ebersberg, Germany). The validation of primers and analysis is consistent with the MIQE guidelines [98,99,100,101,102,103,104,105].

### 4.12. Comet Assay

We performed the alkaline comet assay on gut cells to verify the presence of DNA damage in treated (50 and 100 μg/mL) and untreated OR-R larvae. We followed the standard protocol with some modifications to adapt it to *Drosophila* [106,107]. Third instar larvae were collected and washed in PBS three times. Then, 40 guts per concentration were dissected (two experiments, 20 each) and incubated with 500 μL of 0.25% Trypsin, 2.21 mM EDTA, 1X (Corning cat.25-053-Cl) for 18 min at 40 °C. 

To collect cells, we centrifuged (800× *g*) cell suspension, collected the supernatant, then recentrifuged again (3000× *g*) and recovered the pellet by resuspending in 100 uL of 0.7% low melting point agarose at 37 °C. Then, 50 uL were pipetted on Trevigen CometSlide (2 wells, catalog number 4250-200-03) and kept at 4 °C for 15 min. Then, slides were incubated for 1 h at 4 °C in the dark in a Lysis solution (NaCl 2.5 M, EDTA 0.1 M, Tris 10 mM, DMSO 10%, Triton X100 1%, pH). The alkaline treatment was in the running solution (NaOH 300 mM, EDTA 1 mM, pH) for 20 min, followed by an electrophoretic run for 30 min at 300 mA. Slides were washed three times (5 min each) with neutralization buffer (Tris 0.4 M pH 7.4) and then immersed in 70% ethanol at room temperature for 30 min and stored at 5 °C. The nuclei were stained with ethidium bromide for 5 min (2 μg/mL) and washed two times with dH_2_O. We performed two experiments with two technical replicates. Slides were observed with a fluorescence microscope (ZeissAxio Imager M2) and a 490 nm filter. For each condition, all cells on an area of the comet slide were analyzed with CASP ASP 1.2.3beta2 Image Analysis of Comet Assay.

### 4.13. Carcinogenic Potential 

To assess the carcinogenic potential of PSNPs we exposed *warts* mutant flies following the same exposition protocol as the previous experiments. Parental flies (*wts/TM3 X OR-R*) were left mating on food contaminated with the different NPs concentrations overnight. Then, 3 days old adults were collected, selecting *wts/+* flies based on the absence of the *Stubble* phenotype. The time of NPs’ exposure is about 13 days (from embryo to 3 days old in adults, 10 + 3 days) [108]. Then, we scored an average of 192 *wts/+* flies per concentration, counting the number of tumoral masses and recording their location in the fly bodies (Head, Body, Wing, Leg, Halter).

### 4.14. Statistical Analysis

For the statistical analysis of data, we used Excel and Jamovi (The jamovi project (2024). *jamovi* (Version 2.5) [Computer Software]) to analyze significance. We evaluated the distribution of data with the Sahpiro–Wilk test to decide if to use parametric or not parametric tests based on results. Then, we applied two or one tailed Student *t* test or One way ANOVA (parametric or non-parametric). For statistically significant values, * = *p* < 0.05; ** = *p* < 0.005; *** = *p* < 0.001.

## Figures and Tables

**Table 1:** MPs and NPs effects on in vivo models are resumed in the table. *D. magna* is the most commonly used organism for rapid ecotoxicity screenings, while *D. melanogaster* is the genetic model for excellence. Studies led in those organisms are consistent in showing a decrease in survival and behavioral alterations. *D. rerio* allowed the detection of development outcomes such as delay in hatching and anatomical abnormalities in the juvenile fish. Studies on *M. musculus* reported different and consistent effects on many organs, such as fibrosis, inflammation, and lipid metabolism alterations.

Animal Models	Particle Type and Size	Outcomes	References
*Daphnia magna*	Polystyrene, 53 nm to 200 nm	Reduced life-time and survival, decreased size, and decreased lipid content.	[39,40,41]
*Drosophila melanogaster*	Polystyrene and polyethylene terephthalate, 10 nm to 800 μm	Oxidative stress, genotoxicity, locomotor activity, egg deposition.	[42,43,44]
*Danio rerio*	Polystyrene, 70 nm to 10 μm	Oxidative stress, metabolic disorders, intestinal inflammation, development delay, and abnormalities.	[45,46,47]
*Mus musculus*	Polystyrene, 500 nm to 50 μm	Oxidative stress, metabolic disorders, dysbiosis, hepatic accumulation, inflammation in kidney and liver, reduction of intestinal mucosa.	[48,49,50,51]

## Data Availability

Data is contained within the article and Supplementary Materials.

## Supplementary Materials

The following supporting information can be downloaded at: https://www.mdpi.com/article/10.3390/ijms25147965/s1.
