# Supplementary material for: Plastic Fly: What Drosophila melanogaster Can Tell Us about the Biological Effects and the Carcinogenic Potential of Nanopolystyrene
Source: Int J Mol Sci. 2024 Jul 21;25(14):7965. doi: 10.3390/ijms25147965 (PMC11277132; doi:10.3390/ijms25147965)
Supplement: Supplementary file 1 [file ijms-25-07965-s001.zip › ijms-3097985-supplementary.pdf]

Supplementary Materials

Plastic-Fly: what *Drosophila melanogaster* can tell us about carcinogenic potential and biological effects of nanopolystyrene

Massimo Aloisi, Daniela Grifoni, Osvaldo Zarivi, Sabrina Colafarina, Patrizia Morciano and Anna MG Poma

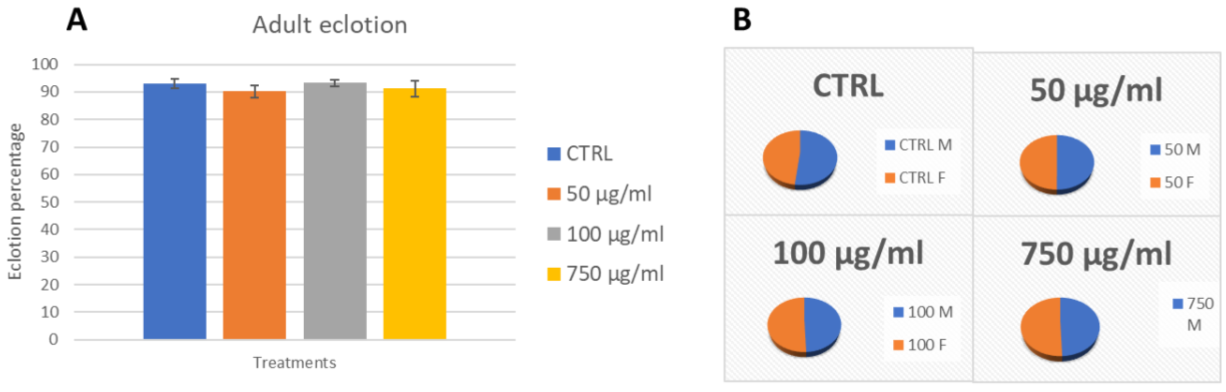

Figure S1. Development traits of exposed adults. Analysis of development traits of OR-R flies: timing of adult eclotion (A) and percentage of females and males hatched (B). No significant results were observed. Treatments: Control (0 µg/ml); 50 µg/ml, 100 µg/ml, 750 µg/ml.

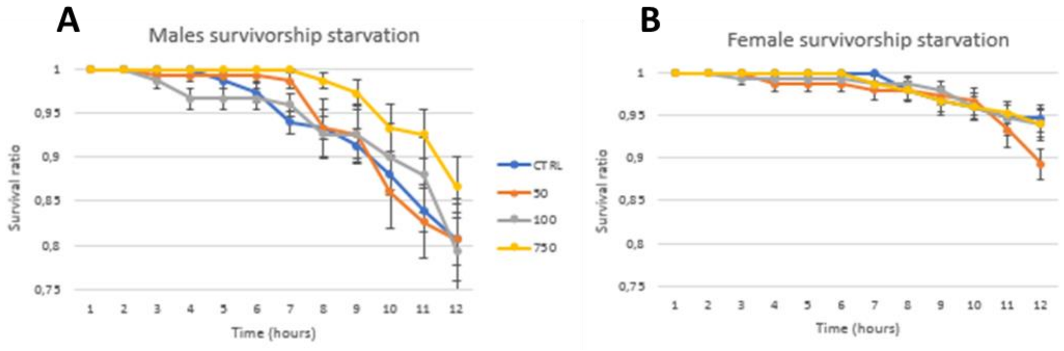

Figure S2. Starvation in exposed adults. Analysis of the survivorship of OR-R flies during the first 12 hours of starvation divided into males (A) and females (B). No significant results were observed. Treatments: Control (0 µg/ml); 50 µg/ml, 100 µg/ml, 750 µg/ml.

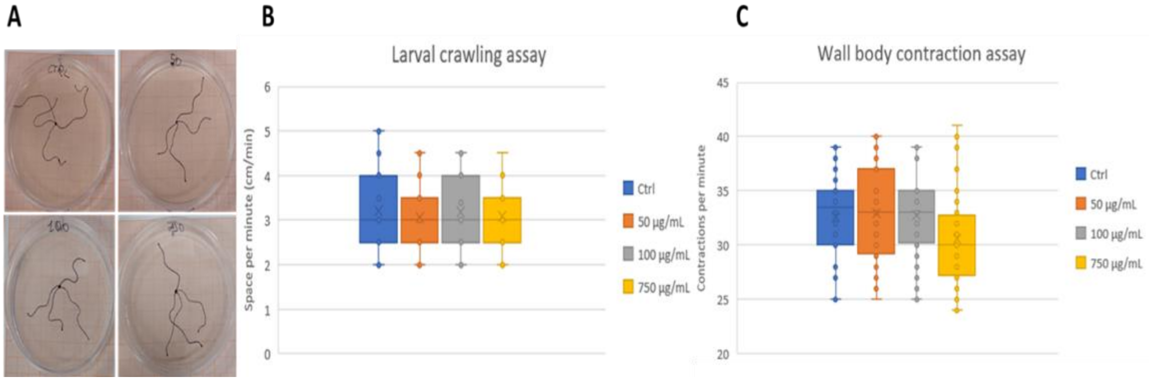

Figure S3. Crawling assay and wall body contraction screening in wild-type third instar larvae fed with PSNPs. A, Petri dishes with 1,5% agarose on a graph paper were used to track larvae paths; B-C, bimodal box plots of crawling assay and wall body contraction screen showing no significant difference between negative control and treatment conditions.

Gene	Primers sequences 5' - 3'	Product size (bp)	Reference
Actin	F: GCGTCGGTCAATTCAATCTT<br>R: AAGCTGCAACCTCTTCGTCA	138	[85]
α tubulin	F: TGTCGCGTGTGAAACACTTC<br>R: AGCAGGCGTTTCCAATCTG	96	[85]
dronc	F: CTCGCTAAACGAACGGAGAAC<br>R: CAACGACACCCACATAAGGG	140	[86]
hid	F: ACGGGCGGCGATGTGTTCTTTC<br>R: TTGGCTGCGGTGTTGATGGC	172	[87]
diap1	F: GTTGCGGCGGTGGTCTCATG<br>R: AGCTTGACGAATCGGCACTGAC	91	[87]

drice	F: CCACTAACAATGGAGAATCCGCC<br>R: GCCGCTGCTACCCGCTCCTC	119	[88]
grim	F: TCGGAGTTTGGATGCTGGGATCTT<br>R: AGTCACGTCGTCCTCATCGTTGTT	153	[89]
rpr	F: AAAGTCCGGCAAATATCGCAAGCC<br>R: TGTTGTGGCTCTGTGTCCTTGACT	196	[90]
p53	F: GCCGCCTCCTTAATCATGCC<br>R: GCCGAGACTGCGACGACTC	133	[91]
rab5	F: GAATCAGGACAGTTTTCAGCGTG<br>R: CTTAGGTAGTTTCTTGGCAATGGC	241	(present study)
rab7	F: AAGTCATCATTCTGGGCGACAG<br>R: CGAGATTCTTGAACGAGTTGGG	270	(present study)

Table S1. Sequence of the primers used for the qPCR analyses.
